# Clinico- pathological profile of patients with breast diseases

**DOI:** 10.1186/1746-1596-8-77

**Published:** 2013-05-09

**Authors:** Hafiz Muhammad Aslam, Shafaq Saleem, Hiba Arshad Shaikh, Nazish Shahid, Anum Mughal, Ribak Umah

**Affiliations:** 1 Dow Medical College, Dow University of Health Sciences, Karachi, Pakistan; 2 Dakshin Dinajpur District, West Bengal, Bangladesh

**Keywords:** Breast diseases, Benign breast diseases, Fibroadenoma

## Abstract

**Background:**

To evaluate the spectrum of breast diseases and their association with presenting complains of patients.

**Methodology:**

It was a cross sectional study conducted from 1^st^ January 2010 – 30^th^ December 2012. A total of 254 breast specimens of patients, who were admitted in Civil Hospital Karachi with breast complaints, were included. Specimens were collected either from mastectomy, lumpectomy or needle biopsy from the admitted patients. Informed written consent was taken from all the patients. All patients with primary breast diseases were included. Patients undergoing chemotherapy or with secondary breast disease and slides with insufficient specimen were excluded. All data was entered and analyzed through SPSS 19.

**Result:**

There were 254 breast lesions, histologically diagnosed in 3 year review period. The overall mean age of patients with breast lesion was 25.18, SD ± 11.73 with a wide age range of 12–74 years. Most common cases identified are benign 191(75.3%), followed by inflammatory 30(11.8%) and malignant lesions 30(11.8%). Most patients presenting with the complain of pain have diagnosis of fibroadenoma 24 (63.2%) while patient with complain of lump also have the most common diagnosis of fibroadenoma 147 (72.8%).

**Conclusions:**

Study shows that in Pakistani females, mostly encountered breast lesion was fibroadenoma. Due to lack of awareness breast diseases present lately. Awareness must be created among women to reduce the mortality and morbidity with breast lesions.

**Virtual slides:**

The virtual slide(s) for this article can be found here: http://www.diagnosticpathology.diagnomx.eu/vs/1037059088969395.

## Introduction

Breast diseases include inflammatory, benign and malignant conditions. Around 200,000 cases of breast diseases are diagnosed annually [1]. Breast diseases are more prevalent among females as compared to males and the pattern of breast diseases and their etiology varies among different countries and ethnic groups [2].

Benign diseases can be classified as inflammatory, epithelial and stroma proliferations, neoplasm and developmental anomalies. Benign breast diseases are more prevalent as compared to malignant and inflammatory, as seen throughout the world [3]. Fibroadenomas are in greater frequency among the population, constituting almost half of all cases of benign diseases [4]. Incidence of benign lesions is common in the second decade reaching on its peak at fourth and fifth decade of life [5]. Risk factors for benign and malignant breast diseases include low parity, nulliparity, low age at first birth and late menopause, highlighting the fact towards excessive circulating estrogen levels [6,7].

Breast cancer is the most commonly diagnosed cancer accounting for 23% of all diagnosed cancers and the most common cause of death in women worldwide. It is the fifth leading cause of death in both sexes combined [8]. Breast cancer is one of the most frequently occurring cancer and cancer related deaths are highly prevalent worldwide, which has become a major public health challenge [9]. After skin cancer, breast cancer is the most common malignancy in women accounting of 16% cancer in women. It has also been present as unknown origin tumor and also one of the first tumors to be ruled out in the presence of tumor of unknown origin in women. Immunohistochemistry is a diagnostic tool for the classification of tumor and up to date several breast markers has been postulate such as beta-catenin, FAK, PIP, MUC1, PSE, e-cadherin, cytokeratin7 (CK7), estrogen and progesteron receptors, gross cystic disease fluid, mammaglobin (MAG)-A and MAG-B [10]. Incidence of malignant lesions is more frequent after menopause [5].

Breast problems for which patients consult doctors are breast pain, nipple discharge and palpable masses. Pathological or physiological nipple discharge is worrisome. 10 to 15% of women with benign breast diseases will complain of pathological nipple discharge. A breast mass and a cyst need histological diagnosis whereas the breast pain (mastalgia) remains the most common symptom in women. A study at Illinois Chicago states that 36% of the women with breast cancer had breast pain only [11].

In Pakistan there are no statistics available on breast diseases hence studies are required to evaluate the incidence and prevalence of breast diseases so that interventions can be done to educate and guide people about the risk factors and management strategies. Though a few studies have been done in Pakistan regarding the breast diseases but still there is a paucity of data through which rising trend of breast disorders can be halted.

Our main objectives were to evaluate the spectrum of breast diseases and evaluate their association with presenting complains of the patient.

## Methodology of breast

It is a cross sectional study, conducted between 1^st^ January 2010 to December 2012. A total of 254 breast specimens of patients, who were admitted in Civil Hospital Karachi with breast complaints, were included. Specimens were collected either from mastectomy, lumpectomy or needle biopsy from the admitted patients. All specimen were taken in 10% buffered formalin and sent to the hispathological department of Dow Medical College for histological analysis. The gross and cut findings were noted and then the samples were placed in respective cassettes and processed in concentration of alcohol, embedded in paraffin and stained over glass slide using Hematoxylin & Eosin dyes. All patients with primary breast diseases were included. However the patients undergoing chemotherapy and secondary breast disease were excluded.

In order to delineate the pattern of breast diseases prevalent in Karachi, histopathological, cytological and frozen section, diagnosis along with presenting complaints and demographic data were collected in a pre-structured questionnaire. Informed written consent was taken from the patients and questions regarding the demographic data and a detailed presenting complains were noted. The specimens collected were classified into inflammatory, benign and malignant breast lesions. Each slide was examined for diagnosis by two pathologists who have experience of greater than 10 years in this field. Any variation in the diagnosis of the specimen by pathologists was confirmed again by the senior most pathologist of the pathology lab and the diagnosis in common was recorded. The study was approved ethically by The Institutional Review Board of Dow Medical College.

All the data was entered and analyzed through SPSS 19. Frequency and percentage were evaluated for categorical data. Mean and standard deviation were evaluated for continuous data.

## Result

There were 254 breast lesions, histologically diagnosed in 3 year review period. The overall mean age of patients with breast lesion was 25.18, SD ± 11.73 with a wide age range of 12–74 years. Most common cases identified were benign 191(75.3%) follow by inflammatory 30(11.8%) and malignant lesions 30(11.8%) (Table 1).

**Table 1:** Represents demographic data and pattern of breast diseases

**S.NO**	**Variables**		**Frequency**	**Percentage**	**Mean age**
1	**Gender**				
		Male	10	3.9	
		Female	244	96.1	
	**Diagnosis**				
2	**Inflammatory lesions**				
		a) Breast abscess	9	3.5	30.77 ± 8.77
		b) Breast ulcer	2	0.8	37.50 ± 10.60
		c) Acute mastitis	7	2.8	26.42 ± 10.65
		d) Chronic mastitis	7	2.8	24.71 ± 6.44
		e) Mammary duct ectasia	1	0.4	35
		h)Granulomatous mastitis	4	1.6	30.26 ± 3.68
		Total inflammatory lesions	30	11.9	
3	**Benign lesions**				
		a) cystic changes	3	1.2	31.66 ± 10.40
		b) fibrosis	4	1.6	35.25 ± 14.31
		c) Fibrosis and adenosis	1	0.4	21
		d) Epithelial hyperplasia	1	0.4	50
		e) papillomas	1	0.4	35
		g) Fibroadenoma	181	71.3	20.56 ± 5.73
		h) Phyllodes tumor	3	1.2	28.66 ± 14.84
		i) Accessory breast	1	0.4	40
		Total benign lesions	191	75.3	
4	**Malignant lesions**				
		b) Invasive ductal carcinomas	30	11.8	45.66 ± 11.63
		Total malignant lesions	30	11.9	
5	**Male breast disease**				
		Gynaecomastia	10	3.9	26.20 ± 10.45

Fibroadenoma is the most common lesion of benign breast disease 181(71.3%) and it occurs mostly in 2^nd^ decade of life with a mean age of 20.56 ± 5.73. Phylloides tumor, the other fibroepithelial tumor 3(1.2%) in the series comprised cases occurring predominantly in the second decade with a mean age of 28.66 ± 14.84 years (Table 1).

Inflammatory lesions accounted for 30 (11.8%) cases. Of these, breast abscess, acute mastitis, chronic mastitis and breast ulcer reported for 9 (3.5%), 7 (2.8%), 7(2.8%) and 2(0.8%) cases respectively (Table 1).

Malignant lesion comprised 30 (11.8%) cases. There were only cases of invasive ductal carcinoma 30(11.8%) which occurred in 4^th^ decade with a mean age of 45.66 ± 11.63 years.

Of the 10(3.8%) cases of Breast diseases encountered in males, all cases were of gynecomastia 10(3.8%). The age range of patients with gynecomastia was 14–42 years, with a mean of 26.20 years ± 10.45years and a peak incidence in the third decade (Table 1).

Mostly cases presented with uni-lateral lesions 191 (75.2%). Most patients came with complain of lump 202(79.5%) and pain 38(15%) in breast (Table 2).

**Table 2:** Represents presenting complains of patients of breast disease

**Serial No**	**Variables**		**Frequency**	**Percentage**
3	**Sides**			
		a) Unilateral	191	75.2
		b) Bilateral	9	3.5
4	**Symptomatology**			
		a) pain	38	15
		b) Breast lump	202	79.5
		c) Nipple retraction	1	0.4
		d) fever	2	0.8
		e) Enlargement of breast	3	1.2

Most patients with complain of pain have diagnosis of fibroadenoma 24 (63.2%) and Invasive ductal carcinoma 5 (13.2%). Patients with presenting complain of breast lump have mostly diagnose with Fibroadenoma 147 (72.8%) and Invasive ductal carcinoma 22 (10.9%) (Table 3).

**Table 3:** Represents relation between diagnosis and complains of patients of breast disease

**Diagnosis**			**Symptomatology**		
	**Breast pain**	**Breast lump**	**Nipple retraction**	**fever**	**Enlargement of breast**
**Breast abcsess**	2 (5.3%)	7 (3.5%)	0 (0%)	0 (0%)	0 (0%)
**Acute mastitis**	2 (5.3%)	6 (3%)	0 (0%)	0 (0%)	0 (0%)
**Chronic mastitis**	2 (5.3%)	7 (3.5%)	0 (0%)	0 (0%)	0 (0%)
**Mammary duct ectasia**	1 (2.6%)	1 (0.5%)	0 (0%)	0 (0%)	0 (0%)
**Fat necrosis**	0	0 (0%)	0 (0%)	2 (100%)	0 (0%)
**Granulomatous mastitis**	1 (2.6%)	4 (2%)	0 (0%)		0 (0%)
**Epithelial Hyperplasia (Proliferative breast disessae without atypia)**	0 (0%)	1 (0.5%)	0 (0%)	0 (0%)	0 (0%)
**Papillomas (Proliferative breast disessae without atypia)**	0 (0%)	1 (0.5%)	0 (0%)	0 (0%)	0 (0%)
**Invasive ductal carcinoma**	5 (13.2%)	22 (10.9%)	1 (100%)	0 (0%)	1 (33.3%)
**Fibroadenoma (Stromal tumors)**	24 (63.2%)	147 (72.8%)	0 (0%)	1 (50%)	2 (66.7%)
**Phyloodes tumor (stromal tumors)**	2 (5.3%)	3 (1.5%)	0 (0%)	0 (0%)	0 (0%)
**Accessory breast tissue**	0 (0%)	1 (0.5%)	0 (0%)	0 (0%)	0 (0%)
**Gynaecomastia**	2 (5.3%)	5 (2.5%)	0 (0%)	1 (50%)	0 (0%)

## Discussion

The incidence of breast disease has dramatically increased over the last decade. Benign breast diseases are a heterogeneous group of lesions, including a variety of tissue abnormalities that are differentially associated with breast cancer risk [1]. With the increase use of mammography more and more, women are diagnosed with benign and malignant breast diseases [12]. Breast carcinoma is now the leading cause of cancer related deaths in women worldwide after lung cancer. In the year 2010 Breast cancer accounted for an estimated 28% of all new cancer cases in United States [13].

Benign breast diseases are the most common breast lesions evaluate in this study. This was also the findings of previous studies [1,4,14,15]. In this study, majority of biopsies which were sent to histopathological department, were of fibroadenomas. These findings reveal high rate of lesions as compare to past studies [1,16-18]. Its peak incidence is in 2^nd^ and 3^rd^ decade of life, but can also occur after menopause due to hormone replacement therapy. These findings were consistent with other studies [19].

In our study Phyllodes tumor accounts for 1.2% of all the breast lesions and have a peak incidence in pre-menopausal age. These findings were comparable with other study also [19]. Phyllodes tumor present histologically as intraductal growth of intralobular stroma with leaf life projections [19].

FIbrocytic change is one of the breast lesions with peak range of incidence at 31–35 years. Our findings were consistent with past study [1]. It occurs during ovulation and just before menstruation. During these times, hormone level changes, which often causes the breast cells to retain fluid and develop into nodule or cyst which feels like a lump when touched. These nodules and cysts spread through out the breast. As hormone level rises just before and during menstruation, mammary blood vessels swell, alveoli expand and cell growth proliferates [1].

There is 11.9% of inflammatory lesion which was much higher as compare to past studies [14-16]. All inflammatory lesions were revealed in early 30s with a mean age from 24.7 ± 6.444 to 30.77 ± 8.77 for mastitis, breast abscess and ulcer. Acute and chronic mastitis constitute 5.8% of breast lesion which is in accordance with past studies [16,20,21]. Similar to observation in all past studies, gynecomastia was the most commonly encounter male breast disease constituting 3.9% of all the cases, as indicate in previous studies [4,20]. Frequency of Gynecomastia was found to be present on its peak in 2^nd^ decade of life, which was earlier as compare to African study [4]. It should not be considered as a disease because enlargement of breast is a common problem. Cause of gynecomastia in most cases is not known, many being idiopathic. It mainly occurs due to excessive estradiol related to testosterone [1]. Higher occurrence may be related to higher incidence of liver cirrhosis following hepatitis B, leading to hyperesterinism and malignancy in susceptible males [18].

Granulomatous inflammatory changes in the breast can be related to specific infectious agents such as Mycobacterium Tuberculosis, non-infectious disease such as sarcoidoses, foreign material as silicon, paraffin or suture material or trauma. It is present in pre-menopausal age. In this study there were four cases of granulomatous mastitis, which was comparable with the past study [1].

Breast cancer is the most common non-cutaneous neoplasia in women. It is a heterogeneous disease, such that it may have different prognostic and therapeutic responses despite similarities in histological types, grade and stage of various subtypes. There are 19 subtypes of breast carcinoma according to World Health Organization (WHO) 2003 classification [22]. Mechanism of developing breast cancer is still unclear but the contribution of mutation in BRCA1 and BRCA2 have been reported to be associated with a dominantly increased risk of disease [9]. Breast cancer susceptibility gene l (BRCA 1) is involved in inhibition of cell growth, cell cycle regulation, gene transcription, DNA damage repair and apoptosis. It was evaluated that dislocation of the cytoplasmic BRCA1 protein in breast cancer cells, is related to the occurrence and metastasis of breast cancer and is expressed in cytoplasm of breast cancer of both younger and elder people [23]. Estrogen, estrone, and estradiol are catabolized to catechol estrogens and their metabolites, such as 4-hydroxyestrone and 4-hydroxyestrone have been involved in breast carcinogenesis. Catechol-O-methyltransferase (COMT) catalyzes the O-methylation of these carcinogenic estrogens to methoxyes tradiols and methoxyestrones. Transition from G to A in COMT gene results in an amino acid change (Val/Met) at codon 108 of soluble COMT and codon 158 of membrane-bound COMT. This shifting in amino acids has been result in a 3–4-fold decrease in enzymatic activity. It has been assesed that individuals who inherit the low activity COMT gene may be at increased risk for breast cancer, because of an increased accumulation of the catechol estrogen intermediates, clearing the pathogenic pathway of developing breast cancer [9]. A principal finding of our study was malignancy rate of 11%, which were consistent with past studies of Pakistan [15,24]. They were not consistent with findings reported in study of Saudi Arabia and Ghana [16,17]. Mean age of its presentation was in 4^th^ decade which was same as indicated in past study [15]. In our study invasive ductal carcinoma becomes the most common and only variety of breast cancer as indicated in past studies [2,24]. Invasive breast carcinoma is associated with a high mortality rate due to invasion in lymph nodes, adjacent tissues and due to metastasis. Invasive ductal carcinoma is the most common histological type with a poor prognosis rate of 30-35% 10 year survival rate. Peritumor lymphatic and blood invasion are the main factors related to presence of metastasis to lymph nodes and they are more closely related to tumor size and histological grade [25].

Most of the patients clinically present with lumps in the breast followed by pain, enlargement of breast, fever and nipple retraction. Our findings were comparable with past studies [11,26,27].

We establish the baseline data for longitudinal study prospective study and current study also adds additional information to the international research literature. Our study was a cross sectional study encircling a small part of the population of Karachi and may not be representative of whole nation.

Breast cancer and breast diseases screening programs should be developed at the hospitals. These programs should ideally include clear objectives, plans and managements. Programs should be free of cost, to encourage large number of women to enroll in such screening programs.

## Conclusion

Benign breast diseases are the commonest breast diseases, in which fibroadenoma is the most common variety. Cancer prevalence is less reported and invasive ductal carcinoma is the most common reported variety. Patients normally present late with locally advanced diseases, due to lack of awareness and knowledge.

## Competing interests

The authors declare that they have no competing interests.

## Authors’ contribution

HMA SS HAS and RU did manuscript drafting and NS, AM did data collection and critically review the manuscript. All authors finalize and agree on final draft. All authors read and approved the final manuscript.
